# Angiolipofibroma of the Cecum: A Rare Type of Submucosal Polyp

**DOI:** 10.1155/2013/737015

**Published:** 2013-05-28

**Authors:** Gabriel M. Groisman

**Affiliations:** Institute of Pathology, Hillel Yaffe Medical Center, 38100, Hadera, Israel

## Abstract

Mesenchymal type tumors originated in the submucosa represent a small percentage of colorectal polyps. This is particularly true for polyps composed of more than one mesenchymal tissue type. We herein present the pathological features of an unusual polypoid proliferation of mature fatty, fibrous, and vascular tissues including vessels of diverse nature and size. The histological findings support a hamartomatous rather than a true neoplastic origin for this rare lesion.

## 1. Introduction

While most colonic polyps are of epithelial nature and originated in the mucosa, a small subset of them represents submucosal proliferations of mesenchymal type cells. The most common types include lipomas, leiomyomas, benign neural and vascular lesions, and gastrointestinal stromal tumors (GISTs) [1]. In the vast majority of the cases, mesenchymal polyps are composed of a single tissue type (e.g., lipoma, hemangioma, and neuroma). In contrast, mixed mesenchymal lesions are extremely rare. We herein present the pathological features of an unusual cecal submucosal polyp composed of adipose tissue, fibrous tissue, and vascular tissue, including lymph vessels and blood vessels of different types and sizes (angiolipofibroma).

## 2. Case Presentation

A 66-year-old male with history of hepatitis caused by HCV presented with abdominal pain of 3-month duration that was not associated with meals or a specific time of the day. The patient had no constipation, diarrhea, or bloody stool. Physical examination revealed no abdominal mass or tenderness. Stool examination was negative for occult blood. The hemoglobin and serum carcinoembryonic antigen levels were within normal limits. A full colonoscopy revealed a smooth-surfaced and sessile round polyp in the cecum measuring 1.0 cm in diameter. The lesion, which was covered by normal-appearing mucosa, was excised using a cold snare. The patient is alive and well with no additional colorectal lesions two years after the polyp's resection.

## 3. Pathologic Examination

Macroscopic examination of the excised specimen showed a circumscribed round polyp, 1.0 cm in diameter, of soft consistency, covered by normal-looking mucosa. Formalin-fixed, paraffin-embedded tissue sections were stained with hematoxylin and eosin (HE) and Weigert's stain for elastic fibers and assessed immunohistochemically for the following markers: CD31 (clone JC70A, dilution 1 : 20; Dako, Hamburg, Germany), D2-40/podoplanin (clone D2-40, 1 : 100; Dako), smooth muscle actin (SMA) (clone 1A4, ready to use, Dako), muscle specific actin (MSA) (clone HHF35, ready to use, Cell Marque, ready to use, Rocklin, CA, USA), desmin (clone D33, 1 : 250; Dako), and HMB-45 (clone HMB-45, Signet, ready to use, Emeryville, CA, USA).

**Figure 1:**
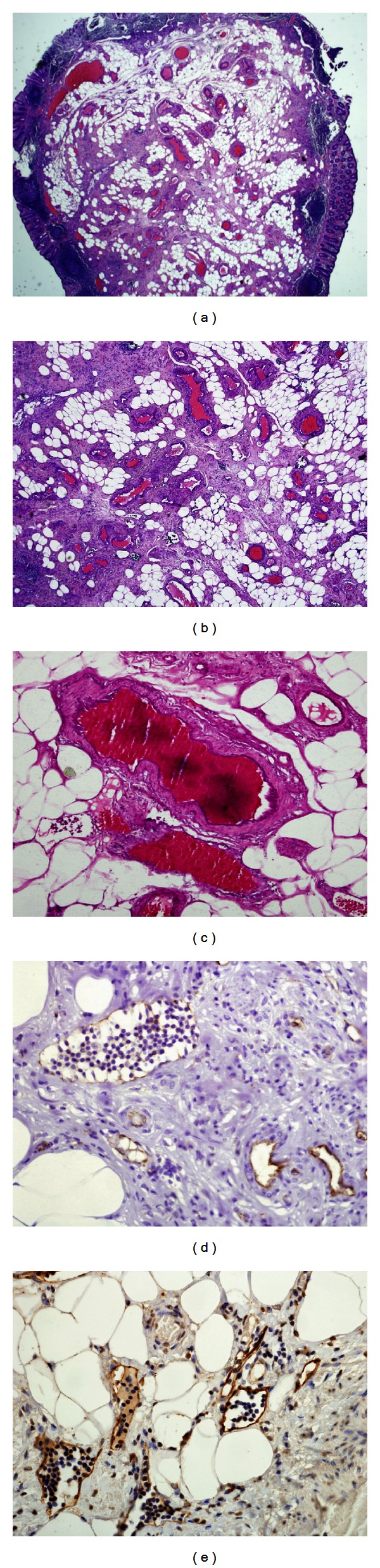
(a) and (b) show a colonic polypoid submucosal proliferation of mature adipose and fibrous tissue with blood and lymph vessels of different sizes (hematoxylin and eosin stained sections, (a) magnification ×20 and (b) magnification ×40). (c) Weigert's stain for elastic fibers highlights the internal elastic lamina in arteries (magnification ×100). (d) CD31 stains endothelial cells from blood and lymph vessels (see large lymph vessel at upper right) (magnification ×400). (e) D2-40 stains the lymphatic endothelium while the blood vessels endothelial cells remain negative (magnification ×400).

 Histologic sections revealed a submucosal proliferation of mature adipose tissue with fibrous septa containing numerous vessels of varied nature (lymphatics, capillaries, small veins, venules, small arteries, and arterioles) lined by a benign endothelial lining (Figures 1(a) and 1(b)). Weigert's stain for elastic fibers highlighted the internal elastic lamina in arteries (Figure 1(c)). No thrombosis or vasculitis was noted. The lesion was covered by unremarkable colonic mucosa containing few lymphoid aggregates. By immunohistochemistry, vascular and lymphatic endothelial cells reacted to CD31 (Figure 1(d)) while D2-40 stained the lymphatic endothelium only (Figure 1(e)). Smooth muscle actin, muscle specific actin, and desmin stained the vessels walls and the muscularis mucosa. HMB-45 was negative.

## 4. Discussion

Since colonoscopy was recognized as the gold standard screening procedure for colorectal cancer, polypoid mesenchymal lesions of the colon are increasingly being detected [2]. In this paper, we describe a submucosal cecal polyp composed of a mixture of mature adipose tissue, fibrous tissue, and vascular structures including lymphatics, veins, arteries, and capillaries. The fact that the lesion was composed of a disorganized overgrowth of mature cells and tissues normally present in the colonic submucosa supports a benign hamartomatous etiology for the lesion and argues against the possibility of a true neoplasm. Angiolipofibromas are exceedingly rare in the gastrointestinal tract. We were able to find two previously reported cases in the English literature. The first was a large pedunculated polyp from the esophagus in a 62-year-old patient that caused dysphagia and was excised surgically [3]. The second was a 12 cm long, finger-like polyp in a 73-year-old male that was originated in the distal duodenum and reached the proximal jejunum. The lesion, which caused recurrent gastrointestinal bleeding, was removed endoscopically [4]. Unlike the previous cases, the present lesion was much smaller and located in the colon. Two additional cases, one in the transverse colon and one in the omentum, were described in the non-English literature [5, 6]. A similar, though not identical, lesion originated in the sigmoid mesocolon and displaying proliferation of vessels of various sizes and types within fibrofatty tissue was described by Demir et al. [7] under the designation of “angiolipomatous mesenchymal hamartoma.” It consisted of prominent vascular channels in a background of fibrofatty tissue that merged with the surrounding mesenteric and retroperitoneal fat. Unlike our lesion, this lesion contained scattered nerves and lymphoid aggregates while no lymph vessels were present. 

 Other entities that may be considered in the differential diagnosis include tumors of adipose and/or vascular tissues of the gastrointestinal tract. Angiolipomas, an important consideration in our case, have been occasionally reported in the gastrointestinal tract. We found 11 cases including one in the esophagus [8], three in the stomach [9–11], eight in the small intestine [12–19], and seven in the colon [20–26]. However, unlike the branching networks of proliferating capillary-type vessels with focal fibrin thrombi, typical of angiolipomas, our case included a mixture of arterial, venous, and lymphatic vessels without fibrin thrombi formation. Angiomyolipoma, another mixed mesenchymal lesion with vascular and adipose components, was reported in 7 occasions in the colon [27–34]. It can be differentiated from our tumor since it contains myoid cells that express smooth muscle markers and HMB-45, the vessels are devoid of elastic fibers, and lymph vessels are not present. 

Less probably to be confused with our case are lesions composed exclusively of adipose or vascular tissue. In fact, the most common lipomatous neoplasms in the gastrointestinal tract are lipomas and their most common location is the right colon. They usually develop as a small, mucosa-covered intramural polypoid lesions [35]. Unlike our lesion, they are composed of uniform, mature adipocytes without proliferation of vessels. Pure vascular lesions seen in the gastrointestinal tract are hemangiomas, lymphangiomas, hemangiolymphangiomas, and vascular malformations, mostly seen in the small bowel and colon [36]. Obviously, all these lesions are devoid of adipose tissue being easily distinguished from the polyp described here.

In summary, we have reported a rare case of submucosal angiolipofibroma of the colon that we believe to be the first reported case in the colorectum in the English literature. This benign lesion appears to be of hamartomatous origin and may be treated successfully by standard polypectomy techniques.
